# The Role of Artificial Intelligence in Herpesvirus Detection, Transmission, and Predictive Modeling: With a Special Focus on Marek’s Disease Virus

**DOI:** 10.3390/pathogens14090937

**Published:** 2025-09-16

**Authors:** Haji Akbar

**Affiliations:** Sharman Lewis School of Agriculture and Applied Sciences, Langston University, Langston, OK 73050, USA; haji.akbar@langston.edu

**Keywords:** artificial intelligence, herpes virus, Marek’s disease virus

## Abstract

Herpesvirus infections, including herpes simplex virus (HSV), Epstein–Barr virus (EBV), and cytomegalovirus (CMV), present significant challenges in diagnosis, treatment, and transmission control. Despite advances in medical technology, managing these infections remains complex due to the viruses’ ability to establish latency and their widespread prevalence. Artificial Intelligence (AI) has emerged as a transformative tool in biomedical science, enhancing our ability to understand, predict, and manage infectious diseases. In veterinary virology, AI applications offer considerable potential for improving diagnostics, forecasting outbreaks, and implementing targeted control strategies. This review explores the growing role of AI in advancing our understanding of herpesvirus infection, particularly those caused by MDV, through improved detection, transmission modeling, treatment strategies, and predictive tools. Employing AI technologies such as machine learning (ML), deep learning (DL), and natural language processing (NLP), researchers have made significant progress in addressing diagnostic limitations, modeling transmission dynamics, and identifying potential therapeutics. Furthermore, AI holds the potential to revolutionize personalized medicine, predictive analytics, and vaccine development for herpesvirus-related diseases. The review concludes by discussing ethical considerations, implementation challenges, and future research directions necessary to fully integrate AI into clinical and veterinary practice.

## 1. Introduction

Infectious diseases pose a substantial challenge to global animal health, particularly in the context of intensive livestock farming [1]. Among these, herpesvirus infections such as herpes simplex virus (HSV), Epstein–Barr virus (EBV), and cytomegalovirus (CMV) represent significant public health concerns worldwide. These viruses are responsible for a wide range of conditions, from mild cold sores to severe complications such as encephalitis, organ failure, and cancer [2]. Despite extensive research, current diagnostic and therapeutic strategies remain insufficient, largely due to the viruses’ ability to establish latency, evade immune responses, and, in some cases, develop resistance to antiviral treatments. This highlights the urgent need for innovative approaches to enhance detection, deepen our understanding of viral transmission, and identify novel therapeutic targets [3,4,5].

In the context of veterinary medicine, herpes virus-associated diseases like Marek’s Disease (MD) are particularly impactful. MD, caused by Marek’s disease virus (MDV), is a highly contagious and oncogenic alphaherpesvirus that affects poultry, especially chickens [6,7]. First described in the early 20th century by Hungarian veterinarian József Marek, the disease manifests in various forms, including paralysis, immunosuppression, and lymphomas, directly impairing weight gain and egg production [7,8]. Indirectly, it contributes to economic burdens through the costs of vaccination and biosecurity measures, with estimated global losses ranging from USD 1 to USD 2 billion annually [9,10]. Although the introduction of vaccines in the 1970s significantly reduced clinical incidence, MDV continues to evolve under vaccine-induced selection pressure, resulting in increasingly virulent strains, including very virulent plus (vv+MDV) pathotypes. These developments underscore the persistent challenge of controlling MDV and the need for adaptive, forward-looking disease management strategies [11,12].

Concurrently, the field of Artificial Intelligence (AI) has undergone rapid transformation, from theoretical computer science models to practical tools in medicine, finance, and agriculture [13,14,15]. In healthcare, AI is now widely used for diagnostic imaging, predictive modeling, and drug discovery [15,16,17,18,19]. AI encompassing several key approaches: machine learning (ML) which refers to algorithms that learn patterns from structured datasets (e.g., mortality rates, SNP data), deep learning (DL), a subset of ML that uses layered neural networks, particularly effective with large unstructured data such as images, sound, or video;, and natural language processing (NLP) which enables AI to extract structured insights from unstructured text such as veterinary reports or research articles) [18,19]. By leveraging these approaches, AI can analyze vast, complex datasets to detect patterns, generate predictions, and optimize decision-making. In veterinary medicine, AI is beginning to redefine how diseases are diagnosed, monitored, and controlled. Applications include automated diagnostic systems, outbreak forecasting, genomic analysis, and optimization of breeding programs. As such, AI holds the potential to revolutionize infectious disease management in animals [20].

This review explores the intersection of AI and veterinary infectious disease management, with a particular focus on MDV. We aim to provide a comprehensive overview of how AI technologies can enhance our understanding of MDV pathogenesis, enable early detection and outbreak control, and support the development of effective and sustainable intervention strategies. By enabling faster diagnostics, more accurate predictions, and improved resource allocation, AI can serve as a cornerstone in modern veterinary epidemiology.

## 2. Overview of Marek’s Disease Virus (MDV)

**Figure 1:**
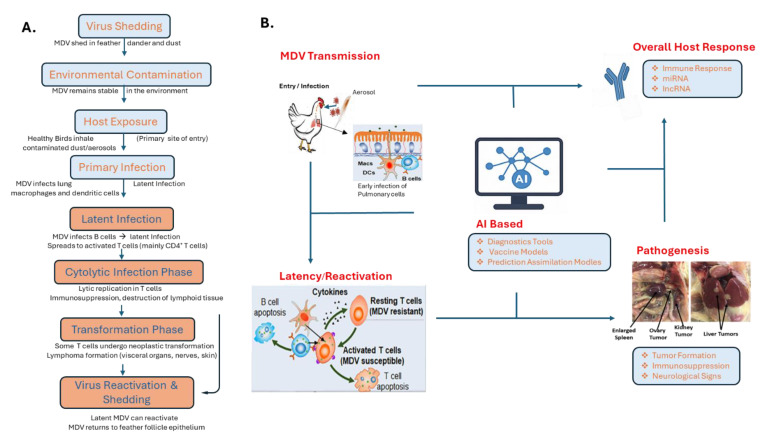
Conceptual overview of Marek’s Disease Virus (MDV) transmission, latency/reactivation, pathogenesis, host responses, and integration of AI tools. (**A**). The figure illustrates the key stages of Marek’s Disease Virus (MDV) infection in chickens, beginning with aerosol-based transmission and viral entry, followed by latency and potential reactivation within host cells. (**B**). Pathogenesis includes tumor formation, immunosuppression, and neurological signs. The host response involves both innate and adaptive immune mechanisms, including regulation by miRNAs and lncRNAs. The figure also highlights the role of artificial intelligence (AI) in enhancing disease understanding, with applications in diagnostics, vaccine development, and predictive modeling.

MDV is an alphaherpesvirus that primarily affects poultry, especially chickens, and is renowned for its oncogenic properties [21,22]. The virus spreads mainly through the respiratory route, either via direct bird-to-bird contact or indirectly through the inhalation of infectious dust and dander. Infection begins in the lungs, where pulmonary macrophages and B lymphocytes capture the virus and transport it to lymphoid organs. This leads to the activation of CD4+ T cells, which subsequently become the primary target cells during the productive lytic phase of infection (Figure 1A) [22,23].

Around 10–14 days post-infection, following a robust immune response, MDV establishes a lifelong latent infection in T cells. Some of these latently infected T cells can undergo malignant transformation, leading to cancer, although this transformation represents a dead end for viral transmission [21,22]. For successful spread within a population, MDV is transported by infected immune cells to feather follicle epithelial (FFE) skin cells, where full viral replication occurs (Figure 1A,B). This results in the assembly of infectious virus particles and their release into the environment [23]. Clinically, MDV infection presents with a range of symptoms including paralysis, ocular changes, immunosuppression, and the development of T-cell lymphomas. Its ability to cause lymphoproliferative disorders and immune dysfunction underscores its significance in poultry health and veterinary medicine [21,22,23,24].

The economic impact of MD is substantial and global. Reports estimate that annual losses due to MDV exceed USD 1–2 billion worldwide [25]. These losses stem from direct mortality, reduced growth rates, decreased egg production, increased carcass condemnation at slaughter, and the financial burden of vaccination and biosecurity measures [9,10]. The emergence of increasingly virulent MDV pathotypes such as very virulent (vvMDV) and very virulent plus (vv+MDV), has further complicated control efforts, as these strains can evade the immunity conferred by current vaccines [11,12].

Despite the widespread implementation of vaccination strategies since the 1970s, MDV continues to pose a significant threat [26,27,28]. Existing vaccines, including HVT (herpesvirus of turkeys), Rispens (CVI988), and MD-Vac (monovalent live virus), provide clinical protection but do not prevent infection or viral shedding [29]. This non-sterilizing immunity creates conditions favorable to the evolution of more virulent strains, a concern highlighted by Read et al. (2015) [28]. The use of such vaccines has altered the virus–host dynamic and raised questions about the long-term sustainability of current control measures [29].

Recent surveillance data show regional variability in MDV prevalence. In developed poultry production systems with strict vaccination and biosecurity protocols, the incidence of clinical MD has declined but not been eradicated. In contrast, areas with poor vaccination coverage or inadequate administration techniques continue to report higher rates of infection [30,31,32]. Additionally, diagnosing MDV remains a challenge due to its clinical similarities with other lymphoproliferative diseases such as avian leukosis and reticuloendotheliosis [33,34,35]. Molecular diagnostic tools, including real-time PCR and next-generation sequencing (NGS), have become crucial for accurate detection and surveillance; however, their application remains limited in low-resource settings [35,36].

Given these challenges, it is firmly established that controlling MDV requires a multi-faceted approach, encompassing robust surveillance, improved diagnostics, and innovative technologies. Among these, AI holds significant promise [37,38]. By integrating complex datasets, recognizing patterns, and predicting outbreaks or vaccine failures, AI could become a powerful tool in the ongoing battle against MDV.

## 3. Role of AI in Infectious Disease Research

### 3.1. Disease Surveillance and Outbreak Prediction

Understanding the transmission dynamics of herpesviruses remains a significant challenge due to their latent nature and asymptomatic shedding [39]. AI-driven epidemiological models, such as agent-based simulations and ML classifiers, offer valuable insights into how herpesviruses spread within populations [40,41]. These models can incorporate a wide range of variables, including environmental conditions, host behavior, and genetic predispositions, to predict outbreaks and identify at-risk populations [41].

AI also plays a crucial role in modeling the effects of interventions such as vaccination programs and antiviral treatments [40,42]. By simulating various scenarios, these models can provide evidence-based guidance on how public health strategies may reduce herpesvirus transmission, particularly in high-risk groups such as immunocompromised individuals [42,43]. One of the most transformative applications of AI in infectious disease management is its capacity for real-time disease surveillance and outbreak prediction [44]. Supervised ML algorithms, including random forests, support vector machines (SVM), and gradient boosting, have been employed to analyze large datasets encompassing climatic, environmental, geographic, and host-specific variables. For instance, features such as temperature, humidity, flock density, and movement patterns can serve as predictive indicators [44,45,46]. Musa et al. (2024) demonstrated the use of mathematical and AI Model techniques in predicting avian influenza outbreaks using key factors demographic, socioeconomic, environmental, and ecological variables [47]. These methodologies can be adapted for MDV surveillance using farm-level data, including vaccination schedules, bird mortality rates, and biosensor outputs.

In poultry farming, sensors continuously collect real-time data on parameters such as temperature, humidity, ammonia levels, and feeding behavior [48]. AI models can process these data streams to detect deviations from normal patterns, which may signal the early onset of an outbreak. Time-series models such as recurrent neural networks (RNNs) and long short-term memory (LSTM) networks are particularly effective for this purpose [49]. For example, Cuan et al. (2022) proposed a new method called “the Deep Poultry Vocalization Network (DPVN)” for the early detection of Newcastle disease based on poultry vocalizations [50]. The method combines multi-window spectral subtraction and high-pass filtering to reduce noise interference and achieves high prediction accuracy within days post-infection. Similar approaches can also be applied to MDV, given appropriate training data.

Another promising advancement is the integration of AI with Geographic Information Systems (GIS) for spatial disease modeling. Predictive mapping powered by AI can identify high-risk zones and inform targeted control measures. This spatial intelligence supports the implementation of focused vaccination efforts and the enhancement of biosecurity protocols in vulnerable areas. For instance, GIS-based reinforcement learning approaches have been utilized to predict disease transmission patterns, aiding in the development of effective intervention strategies [51].

### 3.2. AI for Diagnostic Imaging, Molecular Analysis and Pattern Recognition

AI is rapidly transforming the landscape of diagnostic imaging and molecular analysis, particularly in the detection and monitoring of herpesvirus infections. With advancements in next-generation sequencing (NGS), AI algorithms can now analyze viral genomes to detect herpesvirus strains and mutations that may influence treatment response [52,53]. By evaluating genetic sequences, AI can identify novel variants and track viral evolution, providing insights into changes that may impact pathogenicity or antiviral resistance [54,55].

Traditional diagnostic methods such as polymerase chain reaction (PCR) and serological assays are widely used for herpesvirus detection. However, these approaches can be time-consuming and may miss infections in latent or asymptomatic stages [56]. AI-driven tools, particularly ML models, enhance the sensitivity and speed of diagnostics by integrating and analyzing molecular and imaging data [54,55,57]. In addition, AI algorithms can analyze clinical and diagnostic images such as skin lesions or neurological scans (e.g., MRI and CT) to detect herpesvirus-induced tissue damage. DL models, a subset of AI, are particularly effective at processing large datasets of medical images to identify patterns indicative of viral infection [58]. Similarly, AI-based interpretation of blood samples, genomic sequences, and PCR data can support earlier and more accurate diagnoses, even in cases with minimal clinical symptoms [53].

**Figure 2:**
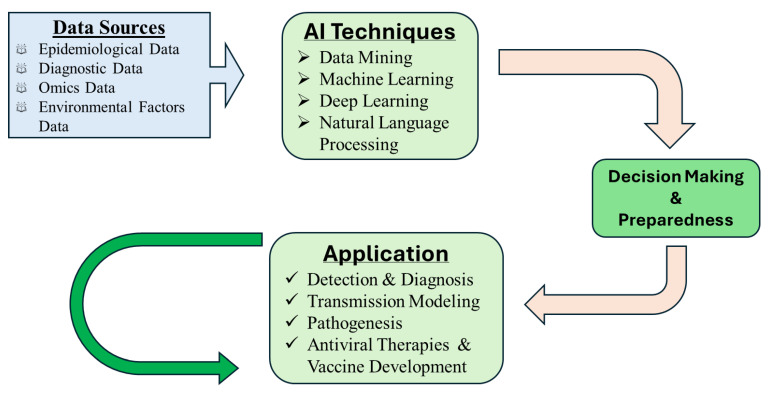
Integrative Framework for AI-Driven Surveillance and Control of Herpesvirus and Marek’s Disease.

In infectious disease diagnostics, image-based evaluations such as gross pathology, histopathology, and radiography remain fundamental. Convolutional Neural Networks (CNNs), a DL technique, have shown exceptional performance in medical and veterinary image analysis [59,60]. These models can be trained to identify subtle yet clinically significant differences between infected and healthy tissue. For MDV, which often manifests as peripheral nerve enlargement and lymphoproliferative lesions in visceral organs [21], AI-assisted image analysis can greatly enhance diagnostic accuracy (Figure 2). For example, digital histopathological slides can be processed by CNNs to quantify lesion severity and distribution. Such as Liu et al. (2019) investigated histologic findings and viral antigen distribution in layer hens naturally coinfected with J avian leukosis virus (ALV-J), MDV, and reticuloendotheliosis virus (REV). Their study utilized fluorescence multiplex immunohistochemistry staining to reveal co-expression of viral antigens in the same tissues and even the same cells, highlighting the complexity of such infections and the potential utility of advanced image analysis techniques in diagnosis [61]. This suggests that AI-driven image analysis, such as CNNs, could be instrumental in quantifying lesion severity and distribution in MDV, enhancing diagnostic accuracy (Figure 2).

This flowchart illustrates the multi-step integration of artificial intelligence (AI) into the surveillance, diagnosis, prediction, and management of herpesvirus infections, including Marek’s Disease Virus (MDV) in poultry. The framework begins with comprehensive data collection from diverse sources, such as genomic data, imaging, sensor outputs, clinical records, and scientific literature. These datasets are fed into AI training pipelines, employing machine learning (ML), deep learning (DL), and natural language processing (NLP) to develop robust models. The resulting AI systems are deployed in various applications, including outbreak prediction, diagnostics, behavioral monitoring, genomic analysis, vaccine response modeling, and selective breeding strategies. Based on AI-driven insights, decisions are made regarding treatment, early warnings, vaccination adjustments, and breeding programs. A feedback loop ensures continual refinement of models through real-world performance data, supporting adaptive and precision-based infectious disease management.

Additionally, AI-enhanced video analytics is emerging as a valuable tool for poultry health monitoring. Video data from flocks can be analyzed to detect subtle changes in behavior or locomotion, such as reduced movement or abnormal gait, which may precede visible clinical symptoms. Recently, Zarrat Ehsan and Mohtavipour (2024) introduced Broiler-Net (https://github.com/TaherehZarratEhsan/Chicken-Behavior-Analysis, accessed on 1 July 2025), a deep convolutional framework designed for real-time analysis of broiler behavior in cage-free poultry houses [62]. The system detects abnormalities such as inactivity and huddling behavior, enabling timely interventions to maintain flock health and productivity. These algorithms support early identification of at-risk birds and contribute to both disease surveillance and animal welfare management.

### 3.3. Genomic and Pathogen Evolution Studies

Genomics is a field where AI has had a particularly transformative impact. NGS-technologies produce vast datasets that require advanced analytical tools for meaningful interpretation [52,63]. AI models particularly unsupervised clustering algorithms and deep autoencoders are capable of detecting patterns in MDV genomic sequences that correlate with virulence, immune evasion, and vaccine resistance [64,65]. ML techniques have been employed to identify single nucleotide polymorphisms (SNPs) associated with pathogenicity. For example, DL tools such as DeepVariant and TensorFlow-based frameworks have been used to annotate viral genomes and predict the functional consequences of genetic variations [66,67]. In the context of MDV, these tools hold promise for detecting the emergence of virulent strains earlier than traditional surveillance methods [68]. Natural language processing (NLP) techniques also play a significant role in genomic and pathogen evolution studies (Figure 2). NLP can extract structured data from unstructured sources, including research articles, diagnostic reports, and epidemiological records [69]. Tools such as BioBERT and SciSpacy enable automated literature mining, accelerating the development of hypotheses related to MDV pathogenesis and control strategies [70].

## 4. AI Applications Specific to Marek’s Disease Virus

AI applications tailored specifically to MDV are beginning to emerge in both research and commercial poultry operations. These applications aim to provide early disease detection, improve vaccine strategies, enhance breeding programs, and ultimately reduce the economic and animal health burden associated with MDV [68,71].

### 4.1. Predictive Modeling of Virulence and Vaccine Breaks

AI-driven predictive modeling is being applied across herpesviruses and avian virology to anticipate virulence shifts and potential vaccine escape events. In MDV, recent synthesis, phylogenomics, field detection of natural recombinants, and in-vivo reverse-genetics delineate virulence-linked polymorphisms (including ICP4 alongside Meq) and evolutionary trajectories that together provide suitable genomic features and labels for future predictive models [31,72,73,74]. In related systems, deep learning predicts EBV integration sites directly from sequence; ML models stratify EBV reactivation risk after hematopoietic stem cell transplantation (HSCT); and ML predicts Newcastle disease virus antigenic distance from F/HN gene sequences—demonstrating feasible pipelines that MDV genomics is poised to leverage [75,76,77].

Beyond genomics, predictive models can also integrate cellular immunity data (e.g., CD8+ T cell frequencies, cytokine responses), humoral immunity (antibody titers), and epidemiological factors (co-infections such as ALV-J or REV, which are known to interact with MDV) [78,79]. Incorporating these heterogeneous inputs enhances the accuracy of predicting vaccine breaks and outbreak risks.

These models can also incorporate field data such as outbreak reports, vaccine strain usage, and farm-level mortality records, to flag regions or farms at risk for vaccine breaks. Tools like XGBoost and LightGBM are used due to their capacity to handle heterogeneous data inputs and highlight key predictive features [80,81]. These insights support regulatory agencies and vaccine producers in adapting immunization strategies and updating vaccine strains in real-time [68,82].

In addition to viral genomic features, host genetics play a critical role in determining outcomes of Marek’s disease virus infection. Several studies have identified quantitative trait loci (QTLs) and single nucleotide polymorphisms (SNPs) associated with resistance or susceptibility to MD [83,84]. Incorporating these markers into AI-driven predictive frameworks, alongside immune response data and epidemiological records, could significantly improve the accuracy of models designed to forecast virulence trends and vaccine breaks [66,72]. By combining viral and host genomic information, predictive modeling can provide a more holistic understanding of MDV dynamics and support targeted intervention strategies.

### 4.2. Early Detection Using Behavioral Data

Behavioral monitoring through AI is gaining traction in commercial poultry settings. Real-time video analytics and wearable sensors track parameters like locomotion, feeding frequency, vocalizations, and thermal imaging data. AI models, particularly CNNs and long short-term memory (LSTM) networks, process these data streams to detect early deviations from baseline behavior [85]. In a case study by Lee et al. (2022), researchers used a DL framework to analyze gait patterns in broiler chickens infected with MDV. The system achieved an 87% accuracy in identifying subclinical infections three days before clinical signs were evident. Early detection allowed farm managers to isolate affected birds, adjust biosecurity protocols, and limit within-flock transmission [86]. These systems not only support disease surveillance but also enhance animal welfare monitoring by enabling real-time intervention in response to stress or discomfort, which often precede overt clinical signs [87].

### 4.3. AI in Understanding MDV Pathogenesis

MDV exhibits several features that make their pathogenesis unique, including their ability to establish lifelong latency and periodically reactivate in response to various stimuli. These properties present significant obstacles to controlling MDV transmission. AI offers an unprecedented opportunity to explore the intricate mechanisms of infection, latency, and reactivation through large-scale data analysis, pattern recognition, and predictive modeling.

Viral Entry and Replication: AI techniques, particularly deep learning, are increasingly being applied to study the interactions between herpesviruses and host cells. Herpesviruses, including MDV, enter host cells through surface receptor binding, followed by membrane fusion and nuclear genome replication [88,89]. Understanding these processes is essential for identifying effective therapeutic targets.

AI-based models can analyze high-throughput drug screening data to identify compounds that inhibit viral entry or replication by targeting specific viral or host proteins. By leveraging large-scale experimental datasets, AI facilitates the discovery of novel antiviral candidates that act at early stages of infection to reduce viral load. Such as AI-powered imaging systems have been developed to detect and classify cytopathic effects caused by various viruses, enabling diagnosis without the need for specific staining [90]. Similar approaches could be adapted to investigate MDV entry and replication mechanisms, supporting the development of targeted antiviral therapies.

Understanding Viral Latency and Reactivation: One of the most critical aspects of MDV pathogenesis is its ability to establish latency within the host, during which the virus remains dormant and evades immune detection. This latent state allows the virus to persist for extended periods and reactivate under certain conditions, such as stress or immune suppression, leading to recurrent infections. AI can be employed to unravel the genetic, epigenetic, and molecular mechanisms underlying viral latency and reactivation, for example, by identifying chromatin modifications and histone demethylase activity (e.g., LSD1) that maintain latency or trigger reactivation. In particular, inhibiting LSD1 has been shown to block α-herpesvirus reactivation, highlighting its role as a candidate marker for reactivation risk [91]. Chromatin state modulation also plays a central role in regulating HSV latency and reactivation cycles [92]. By integrating multi-omics datasets—including transcriptomic and epigenomic profiles, AI and machine learning frameworks can be developed to predict reactivation events. Recent advances in AI-driven epigenetic sequence analysis demonstrate how models trained on histone modification data can predict gene expression patterns, offering predictive power for understanding latency mechanisms [93].

In addition, AI approaches could eventually be applied to model how environmental or immunological factors influence the likelihood of viral reactivation. While such applications remain largely conceptual, mechanistic studies already show that chromatin modifications and histone demethylase activity play central in roles herpesvirus latency and reactivation [91]. These molecular insights provide candidate markers that, once sufficient datasets become available, could be integrated into AI-driven predictive frameworks. Rather than directly simulating reactivation, AI would complement experimental studies by analyzing multi-omics and immune response data to identify regulatory elements that may signal increased reactivation risk. This combined approach could inform the development of novel strategies to prevent viral reactivation and reduce the frequency of recurrent outbreaks, ultimately supporting improved therapeutic interventions for MDV.

### 4.4. AI in Immune Evasion and Viral Persistence

Herpes viruses including MDV have evolved sophisticated mechanisms to evade host immunity, allowing for lifelong persistence within the host. Understanding these immune evasion strategies is essential for developing more effective vaccines and antiviral treatments.

AI in Immune Evasion Mechanisms: MDV employs multiple immune evasion strategies, including the inhibition of antigen presentation, disruption of cytokine signaling, and modulation of host cell apoptosis pathways [94]. AI offers a powerful tool for accelerating the identification and characterization of these mechanisms. By analyzing large-scale proteomic, genomic, and immune response datasets, AI models can predict critical interactions between viral and host proteins involved in immune evasion. For example, prior studies on gamma-herpesviruses have outlined various immune evasion strategies, providing a valuable foundation for AI-based modeling of virus–host interactions [95].

Furthermore, AI can simulate and analyze how MDV proteins interact with host immune cells, enabling the identification of novel therapeutic targets. Such analyses may inform the development of antiviral compounds designed to disrupt these interactions, thereby enhancing the host immune response and improving disease control strategies.

Predicting Viral Persistence and Reactivation Triggers: AI models can also predict what conditions might trigger the reactivation of latent herpesvirus infections. Through the analysis of vast datasets, including infection histories, environmental factors, immune profiles, and genetic predispositions, ML models can identify key patterns that correlate with the risk of reactivation. Although fully integrated multimodal ML models remain limited, foundational studies such as Suzich and Cliffe (2018) elucidate environmental and epigenetic triggers of reactivation at the mechanistic level, while modeling efforts like Canova (2024) demonstrate how machine learning can dissect immune-pathogen interactions influencing latency and reactivation [96,97]. Harnessing this predictive power could be used in veterinary applications such as anticipating when chickens are at higher risk of MDV reactivation and implementing preventive measures, such as antiviral treatment or immune system modulation, to reduce the chances of reactivation.

### 4.5. Enhancing Breeding Programs

Genomic selection for MDV resistance has traditionally relied on QTL mapping and pedigree analysis. However, AI has transformed this field by enabling the integration of multi-omics datasets to identify novel resistance-associated biomarkers. AI models can analyze large-scale proteomic and genomic data to predict interactions between viral proteins and host immune components. In one comprehensive study, researchers used deep neural networks to combine transcriptomic and epigenetic data, successfully identifying key gene networks linked to MDV resistance. Their model highlighted candidate genes such as CD8α and TAP1, both of which are critical in antiviral immune responses [98]. Additionally, the integration of AI-driven genomic evaluations in poultry breeding has been explored as a promising approach to enhance disease resistance traits [93,98]. Incorporating these insights into breeding programs through AI-assisted selection has led to the development of genetically resilient chicken lines with improved resistance to both MDV infection and tumor development [84].

Building on these advances, SNP- and QTL-based approaches provide valuable resources for AI-assisted breeding. Recent studies, such as Lipkin et al. (2024), have mapped genomic regions linked to resistance against MD, demonstrating the feasibility of identifying heritable determinants of susceptibility and resilience [85]. Machine learning models trained on these SNP datasets, especially when combined with transcriptomic or immunological traits, can accelerate the identification of candidate genes and pathways underlying resistance [67,68]. Integrating these genomic insights into breeding programs offers a pathway toward precision selection strategies that enhance flock resilience to MDV while also maintaining productivity and animal welfare standards.

Together, these advancements highlight the potential of AI to drive precision breeding strategies disease resistance without compromising growth performance or other desirable production traits.

## 5. Ethical and Regulatory Considerations

The integration of AI into MDV research in poultry offers significant promise for improving disease surveillance, diagnosis, and vaccine development. However, several ethical and regulatory issues must be carefully addressed to ensure responsible and sustainable implementation.

From an ethical standpoint, the collection and use of data from poultry farms, such as genomic data, production records, and health monitoring information, raise concerns about data privacy, ownership, and informed consent, especially when commercial interests are involved. Ensuring transparency in how farm-level data is collected, shared, and used in AI model training is essential to build trust among poultry producers and stakeholders [99,100,101].

Algorithmic bias is another important consideration. AI models trained on data from specific breeds, production systems, or geographic regions may not perform accurately across diverse poultry populations. To avoid biased outcomes and ensure generalizability, it is crucial that AI systems are trained on comprehensive and representative datasets that capture the variability in genetic backgrounds, farm practices, and environmental conditions [102,103,104].

Economic feasibility must also be considered. Lightweight AI models running on edge devices (e.g., low-power processors, mobile apps) provide practical solutions for resource-limited poultry farms, as they can operate locally without continuous cloud connectivity [105]. While AI infrastructure requires upfront investment (e.g., sensors, cameras, computing devices), the return on investment (ROI) can be substantial through reduced mortality, early outbreak containment, and optimized vaccine use.

On the regulatory side, there is currently a lack of standardized frameworks for the validation and approval of AI-based tools in poultry health. Regulatory authorities and veterinary oversight bodies must develop guidelines to evaluate the accuracy, reliability, and safety of AI applications in MDV diagnosis, outbreak prediction, and vaccine optimization. These frameworks should include requirements for validation in real-world farm settings and continuous post-deployment monitoring to detect unintended consequences [106,107].

Overall, the responsible deployment of AI in MDV research will depend on proactive ethical practices, transparent data governance, and robust regulatory oversight to ensure that these technologies enhance poultry health and support sustainable farming practices.

## 6. Challenges and Limitation in Applying AI to MDV Management

Despite the promising potential of AI in managing MDV and other veterinary pathogens, several challenges limit its widespread application. One major hurdle is the availability and quality of data. AI models depend on large, high-quality datasets for accurate training and validation; however, such datasets are often limited or inconsistent in veterinary medicine. Standardized protocols for data collection and sharing are urgently needed to ensure reproducibility and scalability of AI tools [20]. Another critical issue is the interpretability of AI models. Many advanced models, particularly deep learning algorithms, operate as “black boxes”, offering limited insight into how predictions are generated. This lack of transparency can undermine trust among veterinarians, farmers, and other stakeholders, hindering adoption in real-world settings. Implementing explainable AI (XAI) techniques can enhance understanding and trust in AI-driven decisions [108]. Infrastructure and technical expertise also present significant barriers. The deployment of AI solutions often requires robust digital infrastructure and specialized skills, which may be lacking in certain regions, particularly in low- and middle-income countries. This disparity could widen the digital divide in animal healthcare [109]. Furthermore, the use of AI in veterinary contexts raises ethical and regulatory concerns. Issues related to data privacy, accountability in decision-making, and algorithmic bias must be addressed to ensure responsible and equitable implementation. Establishing clear guidelines and regulatory frameworks is essential for the ethical deployment of AI in veterinary medicine [110]. To address data fragmentation, collaborative data-sharing frameworks such as federated learning have been proposed. Federated learning enables multiple farms or research centers to train models collectively without exchanging raw data, thereby preserving privacy while improving generalizability of AI systems [111]. Such frameworks are already being implemented in human healthcare and could be adapted for veterinary virology [112,113].

## 7. Strategic Directions for Advancing AI in MDV Research and Control

To overcome these challenges and fully harness the potential of AI in MDV research and control, several strategic directions should be pursued. As illustrated in Figure 1, the integration of AI into herpesvirus and MDV management follows a multi-step process, beginning with data acquisition and culminating in actionable decision-making. The flowchart outlines key stages including data collection, AI model training, applications in prediction and diagnostics, informed decision-making, and model refinement through feedback.

Developing open-source, MDV-specific datasets would be instrumental in facilitating AI research and enhancing model performance. These datasets would also foster collaboration and reproducibility across institutions and disciplines. In veterinary medicine, efforts have demonstrated that standardized and accessible data resources improve the quality of medical records and support integrated research across species and institutions [114].

Integrating AI with Internet of Things (IoT) technologies could enable real-time disease monitoring and automated responses, improving early detection and outbreak management. For example, combining AI-powered analytics with on-farm sensors may provide continuous surveillance of flock health and environmental conditions [115]. Advancing AI applications in veterinary medicine will also require close collaboration among veterinarians, data scientists, engineers, and policy-makers. Such multidisciplinary efforts can ensure that AI tools are not only scientifically sound but also practically implementable and ethically grounded. Studies have demonstrated the value of collaborative approaches in enhancing disease identification and preventive care in veterinary contexts [116].

Additionally, the development of explainable AI (XAI) approaches will be crucial for improving transparency and building user trust. By providing interpretable outputs, XAI can help stakeholders better understand and validate model predictions. In clinical decision support systems, explainability allows professionals to comprehend and verify how machine-based decisions are made, thereby augmenting their decision-making processes [117,118]. Continuous updating and validation of AI models using real-world, field-derived data is essential to maintain accuracy and relevance over time. Adaptive models that learn from new data inputs will be better equipped to respond to the evolving nature of MDV and similar pathogens. Retraining AI models with updated data has been shown to enhance their accuracy and effectiveness in dynamic settings [119].

Finally, an important principle is complementarity: AI should not be viewed as replacing traditional veterinary diagnostics or expertise but rather as augmenting them. For example, AI-based histopathology tools still require interpretation by veterinary pathologists; predictive outbreak models inform but do not substitute the judgment of epidemiologists; and sensor-based monitoring provides early warnings that must be verified by farm veterinarians. This synergy ensures responsible and practical deployment of AI in MDV control [120,121].

## 8. Conclusions

AI holds transformative potential in the diagnosis, treatment, and control of herpesvirus infections, including MDV. It enables improved detection, transmission forecasting, drug discovery, and vaccine development, offering a promising pathway toward smarter and more resilient approaches to MDV control. However, practical implementation requires balancing optimism with realism. Significant challenges remain, including data limitations, model transparency, and the need for real-world validation. Importantly, AI should be viewed as complementing rather than replacing traditional diagnostics and veterinary expertise. Future research should prioritize the development of MDV-specific datasets, the adoption of explainable AI to enhance trust, the deployment of lightweight edge solutions for resource-limited farms, and the creation of collaborative frameworks such as federated learning to overcome data fragmentation. By addressing these feasibility challenges while leveraging technological advances, AI can evolve from aspiration to a practical tool for improving poultry health, productivity, and long-term disease resilience.
